# Two cases of vallecular cyst: variable clinical presentation and management outcomes

**DOI:** 10.1093/jscr/rjag648

**Published:** 2026-07-31

**Authors:** Adel Adwan, Isaac Bali, Osama Mohtasib, Ahmad O Amro, Mohammad H Alhousani, Mousa Jaradat, Nasim Abukaresh

**Affiliations:** Department of Surgery, Palestine Red Crescent Society (PRCS) Hospital, Hebron, State of Palestine; Department of Otolaryngology, Faculty of Medicine, Al-Quds University (AQU), East Jerusalem, State of Palestine; Department of Surgery, Palestine Red Crescent Society (PRCS) Hospital, Hebron, State of Palestine; Faculty of Medicine, Al-Quds University (AQU), East Jerusalem, State of Palestine; Department of Surgery, Palestine Red Crescent Society (PRCS) Hospital, Hebron, State of Palestine; Faculty of Medicine, Al-Quds University (AQU), East Jerusalem, State of Palestine; Faculty of Medicine, Al-Quds University (AQU), East Jerusalem, State of Palestine; Faculty of Medicine, Al-Quds University (AQU), East Jerusalem, State of Palestine; Clinical Research, Palestinian Clinical Research Center, Bethlehem, Palestine

**Keywords:** vallecular cyst, infant, stridor, marsupialization, airway obstruction, case report

## Abstract

Vallecular cysts are rare congenital laryngeal lesions. Although histologically benign, they may cause significant upper airway obstruction, particularly in neonates, and can be life-threatening if not promptly managed. We report two cases of vallecular cysts in infants. The first case was a 54-day-old male infant presenting with respiratory distress, inspiratory stridor, and cyanotic episodes. Flexible fiberoptic laryngoscopy revealed a large vallecular cyst. The patient underwent urgent endoscopic excision. Histopathology confirmed a benign cyst lined by respiratory and squamous. The second case involved a 1-year-old male child presenting with chronic snoring, mouth breathing, and feeding difficulties. Imaging demonstrated a well-defined lesion at the base of the tongue. The patient underwent marsupialization of the cyst. Postoperatively, symptoms resolved completely. Vallecular cysts should be considered in infants with persistent stridor or upper airway symptoms. Surgical management is the definitive treatment and associated with excellent outcomes when performed promptly.

## Introduction

A vallecular cyst is a rare laryngeal cyst characterized by mucus accumulation, typically located between the tongue base and the lingual aspect of the epiglottis [1, 2]. The estimated incidence of congenital laryngeal cysts is ~1.8 per 100 000 live births, with vallecular cysts accounting for ~5%–10% of all benign laryngeal cysts [3, 4].

These lesion typically present with respiratory distress, dysphagia, and failure to thrive, and may progress to life-threatening complications, including sudden airway obstruction [5, 6]. The diagnosis of vallecular cysts is primarily established clinically, based on their anatomical location identified during laryngoscopic examination, along with the characteristic cystic features observed on computed tomography (CT) and magnetic resonance imaging (MRI). Histopathological examination remains the gold standard for definitive diagnosis [7, 8]. Surgery is the mainstay of treatment for vallecular cysts [9].

## Case report

### Case presentation 1

A 54-day-old male infant presented with progressive respiratory symptoms, including difficulty in breathing, inspiratory stridor, recurrent choking episodes, post-feeding vomiting, and perioral cyanosis. These symptoms began 1 week after birth and gradually worsened, particularly in the supine position. The patient had multiple prior hospital admissions and was repeatedly treated for upper respiratory tract infections, bronchiolitis, and suspected laryngomalacia without clinical improvement.

The infant was born at term following an uneventful pregnancy and delivery, with a birth weight of 3.3 kg. On admission, he appeared irritable and in moderate respiratory distress, exhibiting suprasternal and intercostal retractions, intermittent tachypnea, and failure to thrive.

Initial investigations revealed mild anemia (Table 1), while chest radiography and arterial blood gas analysis were within normal limits. Echocardiography demonstrated a small patent foramen ovale with left-to-right shunting. Upper gastrointestinal contrast study and cranial ultrasound were unremarkable.

**Table 1:** Complete blood count.

Test	Patient value	Reference range
Haemoglobin (g/dl)	10.8	12–18
White cell count (×10^9^/L)	13.58	4.0–10.9
RBCs (M/μl)	4.03	4.2–6.3
Neutrophils (%)	6.12	40–75
Lymphocytes (%)	43.3	10–58.5
Platelets (×10^9^/L)	401	150–400
Mean corpuscular volume	83.3	80–97

Flexible fiberoptic laryngoscopy (Fig. 1) identified a large, mobile, dome-shaped cyst arising from the right vallecula, resulting in significant supraglottic airway narrowing. Based on these findings, a diagnosis of vallecular cyst was established.

**Figure 1:**
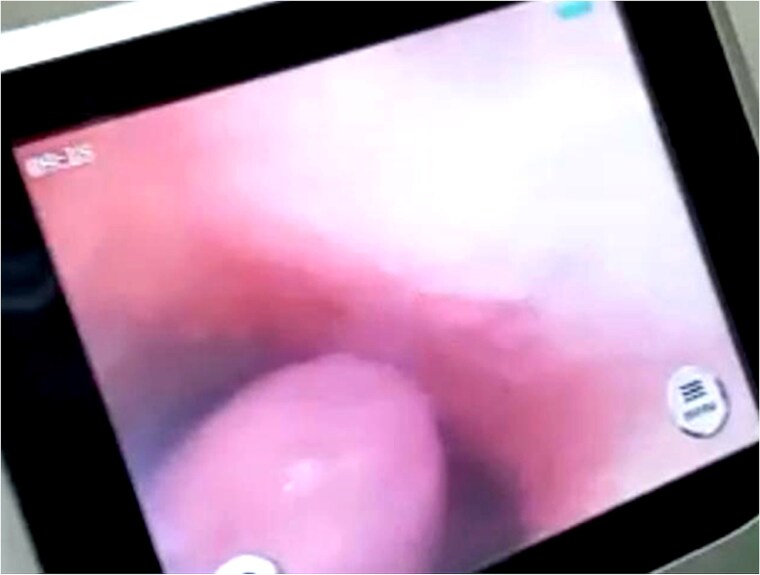
Preoperative flexible fiberoptic laryngoscopy showing a large dome-shaped vallecular cyst causing supraglottic airway obstruction.

The patient was admitted to the pediatric intensive care unit for close airway monitoring. He was kept nil per os and started on intravenous fluids. Urgent direct laryngoscopy under general anesthesia was performed, and complete surgical excision of the cyst was achieved.

Postoperatively, the infant was mechanically ventilated for six hours, then successfully extubated to non-invasive ventilation and subsequently maintained on room air. His respiratory symptoms resolved completely, and oral feeding was resumed without choking or vomiting.

Histopathological examination (Fig. 2) revealed a polypoid cystic lesion lined by both respiratory and squamous epithelium, confirming the diagnosis of a benign congenital vallecular cyst. At follow-up, the patient remained asymptomatic with appropriate weight gain.

**Figure 2:**
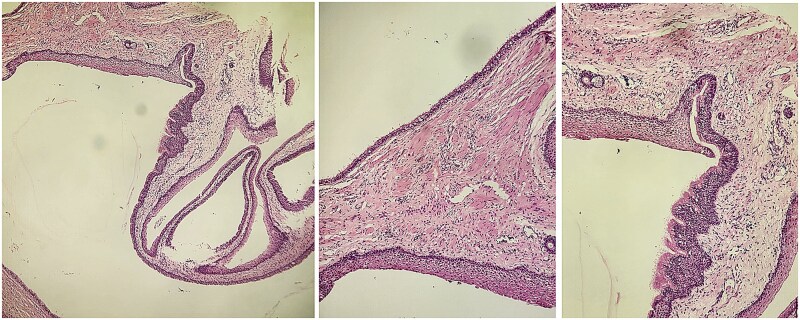
Sections show a polypoid lesion composed of medium-sized glandular and cystic spaces. These spaces are lined by respiratory-type ciliated columnar and squamous epithelium. The intervening stroma is edematous to fibrous with mild chronic inflammatory infiltrate. No cytologic atypia, mitotic activity, necrosis, or infiltrative growth pattern is identified. Negative for malignancy.

### Case presentation 2

A 1-year-old male child presented with persistent snoring and mouth breathing, predominantly during sleep, with gradual progression in severity. These symptoms were associated with feeding difficulties, including poor oral intake and intermittent choking episodes, suggesting possible upper airway compromise.

Notably, there was no history of cyanosis, apnea, stridor, or drooling, making severe acute airway obstruction less likely. There was also no history of trauma or foreign body ingestion. The child remained active, alert, and developmentally appropriate, with no change in activity level. He was not receiving any regular medications.

His past medical history was unremarkable, and surgical history was significant only for right orchidopexy. There were no known drug allergies.

On physical examination, the child appeared well. Skin examination was normal, with no rashes. Ear, nose and throat (ENT) examination (Fig. 3) revealed a soft, cystic, non-tender mass at the base of the tongue, which is a critical clinical finding pointing toward a structural lesion in the oropharyngeal region. The abdomen was soft and non-tender, with no organomegaly. Musculoskeletal and neurological examinations were unremarkable.

**Figure 3:**
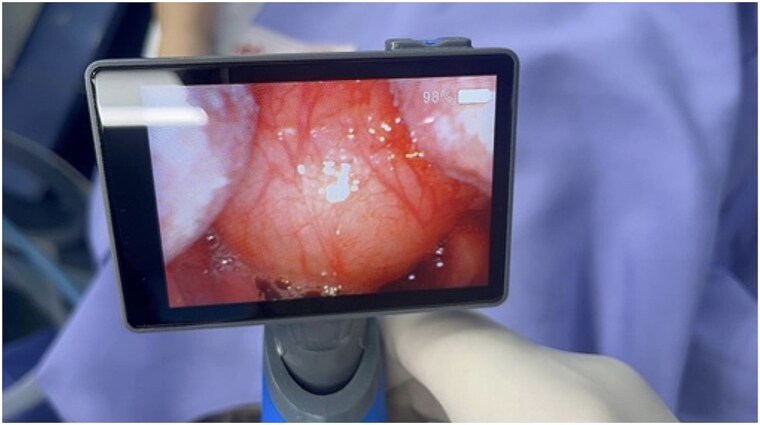
Flexible fiberoptic laryngoscopy shows a mass at the base of the tongue.

Given the clinical suspicion of an upper airway lesion, radiological evaluation was performed. A CT scan of the neck (Fig. 4) demonstrated an ovoid, well-circumscribed midline lesion measuring ~2.5 × 2.2 × 2.2 cm at the base of the tongue above the epiglottis, without evidence of invasive features or significant lymphadenopathy.

**Figure 4:**
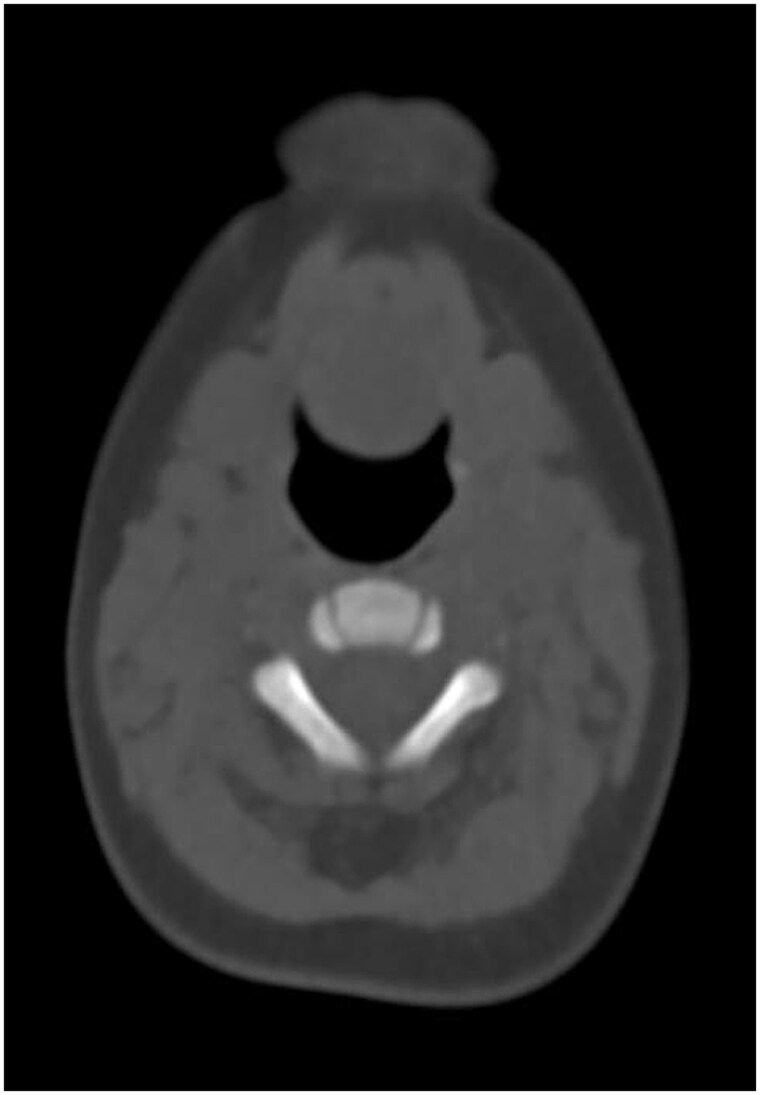
The contrast-enhanced CT of the neck reveals a well-defined, non-enhancing cystic lesion in the midline vallecular space that is causing mild mass effect on the epiglottis and partial narrowing of the airway.

Further characterization with MRI (Fig. 5) provided decisive diagnostic information. The lesion appeared as a well-defined, unilocular cystic structure, measuring ~2.3 × 2.2 × 2.0 cm, hyperintense on T2-weighted images and hypointense on T1-weighted images, with no post-contrast enhancement features that are highly suggestive of a benign cystic lesion. Importantly, the lesion caused a mass effect with posterior displacement of the epiglottis and narrowing of the supraglottic airway, which strongly correlates with the patient’s symptoms.

**Figure 5:**
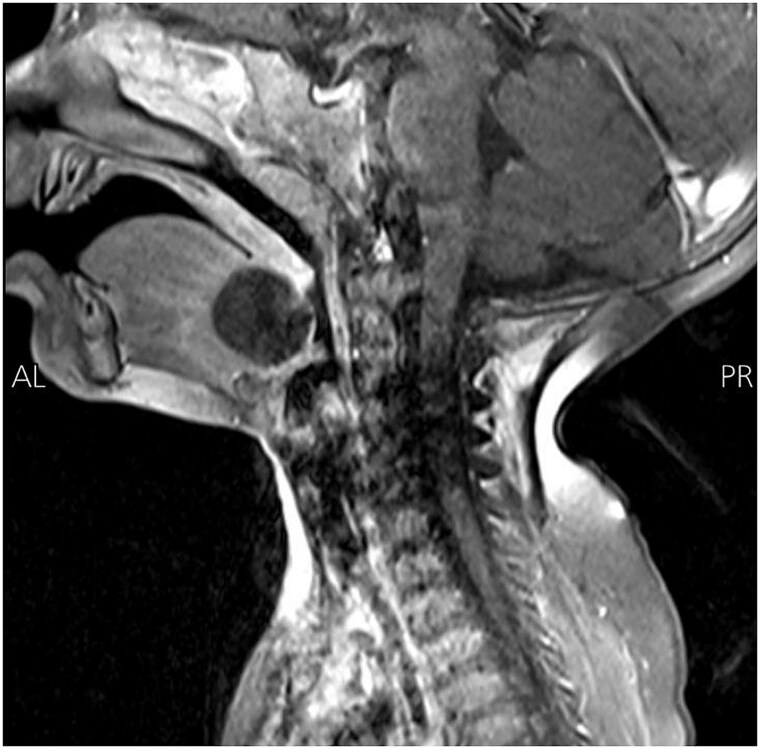
The sagittal MRI reveals a large, well-defined, hypointense cystic lesion within the vallecula that is causing significant narrowing of the oropharyngeal airway.

The combination of a midline cystic lesion located in the vallecular region, characteristic MRI signal pattern (T2 hyperintensity, T1 hypointensity, no enhancement), and mass effect on the epiglottis is highly indicative of a vallecular cyst, effectively narrowing the differential diagnosis. Alternative considerations such as cystic ranula or lingual thyroglossal duct cyst were less likely due to the precise anatomical location and imaging characteristics.

The patient was admitted for definitive management and underwent marsupialization of the vallecular cyst under general anesthesia, which is the standard treatment for such lesions. Postoperatively, he was monitored in the pediatric intensive care unit.

Histopathological examination revealing a cyst lined by respiratory-type ciliated columnar epithelium with areas of squamous epithelium, along with an edematous to fibrotic stroma and mild chronic inflammatory infiltrate. Crucially, there was no evidence of atypia, mitosis, or malignancy, confirming a benign vallecular cyst (Fig. 6).

**Figure 6:**
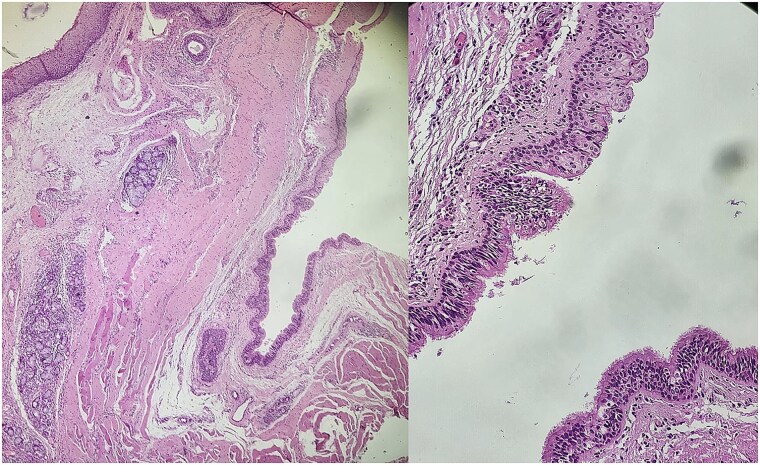
Histopathological examination (H&E stain) of the excised lesion demonstrating a benign vallecular cyst lined by respiratory-type ciliated columnar epithelium and focal areas of squamous metaplasia, supported by an edematous to fibrotic stroma with mild chronic inflammatory infiltrate and no evidence of atypia or malignancy.

The postoperative course was uneventful. The patient remained hemodynamically stable, maintained adequate oxygenation on room air, and demonstrated normal neurological status. Feeding was gradually reintroduced and well tolerated.

Clinically, the child showed marked improvement, with resolution of snoring and feeding difficulties, restoration of normal oral intake, and a clear airway. He was discharged in stable condition without complications.

## Discussion

Vallecular cysts are rare congenital lesions of the larynx. Their development is thought to occur through two main mechanisms: the ductal obstruction theory, in which blockage of mucous gland ducts leads to mucus retention and cyst formation, and the embryologic maldevelopment theory, which involves persistence of epithelial remnants during laryngeal development. Although these cysts are histologically benign, their location at the base of the tongue places them in close proximity to the supraglottic airway, making them potentially life-threatening particularly in neonates and young infants [10, 11]. Histopathological examination confirms the diagnosis, usually revealing a cyst lined by respiratory epithelium with possible squamous metaplasia, along with occasional chronic inflammatory changes due to irritation or infection [2]. In the present cases, histopathological examination showed cysts lined by respiratory epithelium with areas of squamous metaplasia, consistent with benign vallecular cysts. The absence of dysplasia or malignancy further supports the benign nature of vallecular cysts.

Newborns with vallecular cysts typically present with inspiratory stridor accompanied by varying degrees of upper airway obstruction, which may include apnoea, cyanosis, a hoarse cry, and failure to thrive [12, 13]. Life-threatening episodes of airway compromise are not uncommon in this age group. Most infants are diagnosed early in life, usually between birth and 16 weeks of age, with a mean age at diagnosis of approximately 40 days. With time, ongoing mucus production can contribute to cyst enlargement, resulting in progressive worsening of swallowing dysfunction, aspiration, and increasing airway obstruction [14]. Our first case closely paralleled this classic pattern reported in the literature. The patient presented at 54 days of age with progressive inspiratory stridor, recurrent choking, cyanotic episodes, post-feeding vomiting, and failure to thrive, which are all consistent with the severe obstructive phenotype described in young infants. In contrast, the second case differed from the typical neonatal presentation, as the child was older and had a more chronic and compensated course characterized mainly by snoring, mouth breathing, and feeding difficulty without cyanosis, apnea, or acute respiratory distress. This comparison highlights that vallecular cysts may present along a clinical spectrum, ranging from early life-threatening airway obstruction to delayed sleep-related and feeding symptoms.

This report highlights the bimodal clinical presentation of vallecular cysts. The neonatal case showed a severe and rapidly progressive course with stridor, cyanosis, feeding difficulty, and failure to thrive, while the older infant presented with a more chronic and compensated pattern, mainly sleep-disordered breathing and feeding problems without acute airway obstruction. This variability depends on factors such as cyst size, growth rate, degree of supraglottic obstruction, and its interaction with the epiglottis during breathing. Importantly, the neonatal case also emphasizes a diagnostic challenge, as vallecular cysts are often initially misdiagnosed as laryngomalacia. This was also observed in our first patient, who had multiple previous admissions and was treated for upper respiratory tract infections, bronchiolitis, and suspected laryngomalacia before the correct diagnosis was established. This clinical course is consistent with previous reports showing that vallecular cysts may mimic more common causes of infant stridor, thereby delaying definitive treatment.

The definite diagnosis of vallecular cysts is mainly established by flexible fiberoptic laryngoscopy, which allows direct visualization of the lesion, assessment of the degree of airway obstruction, and guidance for airway management [14, 15]. Imaging studies serve as an important adjunct, particularly in atypical or delayed cases. CT scan helps define the size and extent of the lesion, while MRI provides superior soft-tissue characterization, typically showing T2 hyperintense and T1 hypointense cysts without contrast enhancement [2]. Imaging is also valuable for excluding other midline cystic lesions such as lingual thyroglossal duct cysts, dermoid cysts, and ranulas, which differ in surgical management and recurrence risk. The diagnostic work-up in our two patients reflects this approach. In the first case, flexible fiberoptic laryngoscopy was sufficient to identify the obstructing vallecular cyst and guide urgent airway intervention. In the second case, because the presentation was more chronic and the lesion appeared as a base-of-tongue mass, CT and MRI were particularly useful in confirming the cystic nature of the lesion, defining its relationship to the epiglottis and supraglottic airway, and narrowing the differential diagnosis. Histopathological examination confirms the diagnosis, usually revealing a cyst lined by respiratory epithelium with possible squamous metaplasia, along with occasional chronic inflammatory changes due to irritation or infection [2]. Similarly, histopathology in our cases showed respiratory-type epithelium with areas of squamous epithelium or squamous metaplasia, supporting the diagnosis of benign vallecular cyst and aligning with previously reported pathological findings. The absence of dysplasia or malignancy further supports the benign nature of vallecular cysts.

Definitive management of symptomatic vallecular cysts is surgical. Endoscopic marsupialization is widely used and is often considered the standard approach [5, 16], particularly for broad-based cysts; however, complete endoscopic excision is also an accepted treatment option when the lesion is well circumscribed, mobile or pedunculated, safely accessible, and can be removed without excessive risk to adjacent supraglottic structures. Therefore, the operative technique should be individualized according to cyst size, site of attachment, degree of airway compromise, intraoperative exposure, and surgeon judgment. This individualized approach explains the different surgical strategies used in our two cases. In the first case, complete excision was selected because the cyst was large, mobile, and associated with significant airway compromise in a young infant with recurrent cyanotic episodes. In the second case, marsupialization was chosen for a larger midline vallecular cyst presenting with a more chronic clinical course. The complete resolution of symptoms in both patients is consistent with the favourable outcomes reported in the literature after definitive endoscopic treatment. These cases therefore support the view that both excision and marsupialization can be effective when selected according to cyst morphology and clinical severity. In our first case, complete excision was performed because the neonate had severe and progressive airway symptoms, including recurrent cyanotic episodes, and the cyst was large, mobile, and amenable to safe removal under direct laryngoscopy. In the second case, marsupialization was selected for a larger midline vallecular cyst with a more chronic clinical presentation. Both approaches resulted in complete symptom resolution without postoperative complications or recurrence during follow-up.

## Conclusion

Congenital vallecular cyst, although rare, represents an important and potentially life-threatening cause of upper airway obstruction in infants. Clinical presentation is variable, ranging from acute respiratory distress with feeding difficulties in neonates to more insidious symptoms such as snoring and dysphagia in older infants, which may lead to delayed diagnosis or misdiagnosis. Early recognition through flexible fiberoptic laryngoscopy, supported by radiological imaging when needed, is essential for accurate diagnosis and timely intervention. Surgical management, either complete excision or marsupialization, provides definitive treatment with excellent outcomes and minimal risk of recurrence with excellent outcomes and minimal risk of recurrence. These cases emphasize the importance of maintaining a high index of suspicion for vallecular cysts in infants with unexplained respiratory or feeding symptoms to prevent potentially fatal airway compromise.
